# Victimization and Peer and Parents Attachment: The Mediating Effect of Regulatory Emotional Self-Efficacy

**DOI:** 10.3390/ijerph18042062

**Published:** 2021-02-20

**Authors:** Paula Samper-García, Elisabeth Malonda-Vidal, Anna Llorca-Mestre, Roger Muñoz-Navarro, Vicenta Mestre-Escrivá

**Affiliations:** 1 Department of Basic Psychology, University of Valencia, 46010 Valencia, Spain; elisabeth.malonda@uv.es (E.M.-V.); anna.llorca@uv.es (A.L.-M.); maria.v.mestre@uv.es (V.M.-E.); 2 Departament of Psycology and Sociology, University of Zaragoza, 50009 Zaragoza, Spain; rogermn@unizar.es

**Keywords:** victimization, peer, parents, attachment, regulatory emotional self-efficacy, adolescents

## Abstract

Studies of the Spanish adolescent population has concluded that victimization is related to lack of emotional regulation and impulse control. Therefore, if a victim is unable to recognize, understand and regulate their emotions, this can result in rejection by their peers. A cross-sectional study was conducted to examine regulatory emotional self-efficacy as a possible mediator in the association between peer and parents attachment and victimization. Adolescents (*n* = 563) completed Regulatory Emotional Self-Efficacy, Inventory of Parents and Peer Attachment and Kid at School questionnaires. Structural equation models (SEMs) were used to predict a latent variable of victimization with parents and peer attachment, emphasizing the mediating role of regulatory emotional self-efficacy, as comprised by a positive and a negative aspect. Results showed that peer attachment had an indirect negative effect, through perceived self-efficacy, in managing a positive effect in victimization, while father attachment had an indirect negative affect, through perceived self-efficacy, in managing a negative affect in victimization, and Mother attachment had no statistically significant indirect effect in victimization. This study suggests that the roles of parents and peers, and also between mothers and fathers, are different in relation to the perception of victimization of adolescents. Findings provide relevant information regarding implications for prevention and intervention in victimization.

## 1. Introduction

Victimization is defined as the experience of being the object of physical, verbal or psychological aggression behaviors perpetrated by peers in the school environment, especially in places with low supervision by adults. It implies repeated exposure to hostile behavior by schoolfriends [1,2,3].

The seriousness of this problem makes it essential to try to prevent its appearance. To this end, it is necessary to identify the variables as risk or protection factors which allow prediction of said appearance. This study provides an explanation of some of the variables related to this problem, including peer and parents attachment and emotional self-efficacy.

### Literature Review

Social relations among school friends change from the beginning of adolescence to early adulthood. They gradually gain more importance as the bonds with peers begin to satisfy the adolescent need for attachment. It is then when there is a significant rise in peer attachment [4], whereas, during childhood, these needs are normally satisfied by the parents. By family attachment we mean the emotional lasting bond that the child develops with a particular attachment figure [5,6], who strengthens the child giving them emotional security and stability [7] and is related to positive emotional experiences [8]. In this sense, adolescence marks a period of transition during which the dependence on the relationship with the parents changes to dependence on the relationships with schoolfriends [9,10]. During this period, social relationships with peers gain special importance due to the relevance they have for the adolescent, and also because of the impact they have on the psycho-social adjustment of the person. In this sense, the acceptance and attachment by schoolfriends is essential for socialization, internalization of values and development of social skills [11,12]. In general, the peer group gives security and emotional support. It constitutes a safe haven of proximity [13], and all of it contributes to a greater psycho-social adjustment [14]. On the other hand, there are studies which demonstrate the relationship between parental affect, especially the attachment established with the mother, and peer attachment during adolescence. Therefore, boys and girls who formed a safe bond in their family during childhood tend to maintain this same kind of attachment in their relationship with peers [4]. In the present study, both parent attachment and peer attachment are assessed.

Close relationship with peers, and a positive family environment, can be protection factors against aggressive behavior and victimization, and can even lessen the effects that low empathy has as an enhancer of behavior to harm others [15]. It has been confirmed that a safe family attachment relates positively with the ability to self-regulate one’s emotions in both early [16] and mid childhood [17]. In this context, acceptance and attachment with peers is associated with the ability to perceive emotion and problem solving. Thus, emotional regulation has been identified among the emotional variables that help the child and the adolescent to establish relationships and, therefore, to adapt socially [9,18,19]. Free-flowing relationships with schoolfriends tend to be prosocial and offer an environment in which children can practice their self-regulation and self-control skills [17,20].

Emotional regulation and self-control are constructs which have been studied in relation to interpersonal relationships and adaptive and maladaptive behaviors in difficult situations, which require a solution to a problem on the part of the subject. Studies on emotional intelligence confirm the importance of self-awareness and emotional self-control when facing stressful situations. Furthermore, they are considered central processes for psychological resilience in the long-term effects of multiple negative vital events [21,22] such as could be bullying and victimization. It has been demonstrated that coexisting with school friends provides unique opportunities for self-regulation and emotional connection [23], and several studies provide empirical evidence that emotion regulation and coping are closely related [17,24,25].

Related to this, according to Bandura’s self-efficacy theory [26], Caprara [27] developed the concept of regulatory emotional self-efficacy, which includes two dimensions: self-efficacy in managing negative affect, and self-efficacy in expressing positive emotions. We based this study on this concept to assess the role of the belief in one’s ability to manage positive and negative emotions.

Recent research on victimization has focused on cognitive and emotional processes as regulating mechanisms, and mechanism of control of aggressive behavior and victimization among peers [28,29,30]. Social skills, strategies to face and solve problems, emotional intelligence or self-control in the interaction with peers are considered protection variables or, in the case of their absence, vulnerability variables, for good social adaptation and good peer attachment [31,32,33].

Other studies have concluded that victimization is related to a lack of emotional regulation and impulse control [24,34,35]. This way, if the victim is incapable of recognizing, understanding and regulating their emotions, this can provoke rejection in their schoolfriends. In general, the inability to recognize, understand, manage and express their own emotions and those of their school friends, can be a strong predictor of a possible victim [35].

In the last decades, many studies have explored the mediator role of self-efficacy and especially of emotional self-efficacy [24,36,37,38]. These studies brought to light, among other things, the role of emotional self-efficacy between the different variables and victimization. Specifically, self-efficacy dimensions were found to moderate the relationship between victimization and coping [24]. In this study, students with high emotional self-efficacy reported lower use of passive avoidance to cope with almost all forms of victimization. On the other hand, it has been demonstrated that emotional self-efficacy mediates the association between self-esteem and victimization [38], so that the reason why students with a low-level of self-esteem engage in more bullying behaviors may be due to their low level of emotional self-efficacy.

Taking as a basis the previous research, the aim of this study was to examine the role of regulatory emotional self-efficacy as a possible mediator in the association between peer and parents attachment and victimization.

We hypothesized that peer and parents attachment could influence victimization through regulatory emotional self-efficacy. In this sense, there would be a positive relationship between peer and parents attachment with emotional self-efficacy, and a negative relationship of this with victimization.

## 2. Materials and Methods

### 2.1. Participants

563 adolescents (313 boys and 250 girls; M_age_ = 12.73 years, SD_age_ = 0.78), ages 12–15 years, completed the survey and comprised the sample, in Valencia (Spain). Adolescents were in the first year of secondary school. Participating schools were randomly selected from the list of all schools in Valencia with students enrolled in compulsory secondary education. In total, nine schools participated in the study.

Regarding family structure, 70.8% of adolescents came from two-parent households where parents were married, and 23.2% of them in which parents were divorced. In relation to the educational level, 24% of mothers had less than a secondary school diploma, 35.4% had a secondary school diploma or equivalent and 40.6% had some university education. Furthermore, 30.3% of fathers had less than a high school diploma, 35.2% had a high school diploma or equivalent and 34.6% had some university education. Regarding origin, 89.7% of students reported being from Spain. Small percentages of the remaining adolescents self-identified themselves as being from Latin America and eastern European countries. The characteristics of survey participants are described in Table 1.

### 2.2. Research Procedure

The research was approved by the Valencian Government. Parental consent and approval from the School Council were obtained. Participation by adolescents was voluntary; adolescents were free to decline to participate. The survey was administered by trained researchers in the classroom in 50-minute sessions during school hours. The study followed all ethical guidelines pertaining to studies carried out on human beings included in the Helsinki Declaration, under current regulations, respecting respondents’ anonymity for both data collection and data analysis. This study had a favorable response from the University Ethics Committee because it is required for the concession of Research Projects.

### 2.3. Instruments

Victimization was evaluated with the questionnaire Kid at School (KS) [39]. This scale evaluates perceived victimization in three subscales: relational victimization (Cronbach’s alpha of original scale = 0.84) refers to behaviors that are intended to harm through “intentional manipulation and damage of the relationship between peers”; overt victimization (Cronbach’s alpha of original scale = 0.89) includes physical behaviors such as hitting, and verbal, such as insults, intended to harm others; and social exclusion (Cronbach’s alpha of original scale = 0.86). The total score of the scale is the mean of the items score. Students have to answer on a Likert scale of five response alternatives (1 = “almost never”, 3 = sometimes, and 5 = “almost always”). Examples of items are “How often do classmates in your center make fun of you or insult you?”; “Do they tell lies, gossip, spread rumors or spread bad news about you?”; “Do they leave you out of conversations, games or activities?” Cronbach’s alpha for this research was 0.96. 

Parents and Peer Attachment variables were assessed with the Inventory of Parent and Peer Attachment (IPPA) [40]. This instrument evaluates behavioral and affective/cognitive dimensions, communication, trust, and alienation, related to peer, father and mother attachment. In the original scales, the internal consistency (Cronbach’s alpha) for the parent scales was trust (alpha = 0.91), communication (alpha = 0.91) and alienation (alpha = 0.86). For the peer scales they were trust (alpha = 0.91), communication (alpha = 0.87) and alienation (alpha = 0.72). Examples items are, “My friends respect my feelings” and “My mother/father helps me to understand myself better”. Cronbach’s alpha for this study was 0.79 (total peers), 0.89 (total father), and 0.86 (total mother).

Perceived self-efficacy to express positive affect and Perceived self-efficacy in regulating negative affect were assessed with the Regulatory Emotional Self-Efficacy Scale [41]. This scale evaluates self-efficacy beliefs in the domain of emotion regulation using a five-point Likert scale ranging from being unable to fully capable. Two scales were included: (a) perceived self-efficacy to express a positive affect was measured by five items in terms of perceived ability to express liking and affection toward others, to get oneself to express enthusiasm and enjoyment and to feel satisfaction with personal accomplishments. A sample item was ‘‘I can show liking for a person toward whom I am attracted”. Perceived self-efficacy in regulating a negative affect was assessed by nine items in two subscales: (b) perceived self-efficacy in managing anger/irritation assessed the perceived ability to manage a negative affect in the face of anxiety-arousing threats, anger provocation, rejection and disrespect, and to control worrisome ruminations when things go wrong (“I can manage negative feelings when reprimanded by my parents or significant others”); and (c) perceived self-efficacy in managing despondency/distress measured the perceived ability to manage a negative affect in the face of despondency and discouragement (“I can keep from getting discouraged in the face of difficulties”). The total score of each scale was the mean of the items score. Cronbach’s alphas for original scales were for (a), (b) and (c) 0.85, 0.73 and 0.85 for Italians; 0.69, 0.70 and 0.72 for the U.S. sample and 0.64, 0.68 and 0.81 for Bolivians. Cronbach’s alpha for this study was 0.74 (perceived self-efficacy to express positive affect) and 0.80 (perceived self-efficacy in regulating negative affect).

The data about items composition and reliability is shown in Table 2.

### 2.4. Data Analysis

SPSS 26 was used to analyze means and standard deviations and to calculate correlation analysis to test the relations among variables. Furthermore, structural equations modeling (SEM) in Mplus 6.1 [42] was employed to explore a cross-sectional model. The following goodness-of-fit indexes were used: chi-square divided by degrees of freedom (χ^2^/d.f.), Bentler comparative fit index (CFI) and Tucker–Lewis Index (TLI). Root mean square error of approximation (RMSEA) was used to measure error [43]. Finally, indirect effects were tested using the bias corrected bootstrap confidence interval method in Mplus [44,45].

## 3. Results

### 3.1. Descriptive Statistics, and Correlational Analyses

Table 3 presents means, standard deviations and results for the correlations. The correlations showed that the victimization is significantly negatively correlated to peer attachment and regulatory emotional self-efficacy (perceived self-efficacy to express positive affect, and perceived self-efficacy in regulating negative affect) (Table 3).

### 3.2. Structural Equation Model

**Figure 1:**
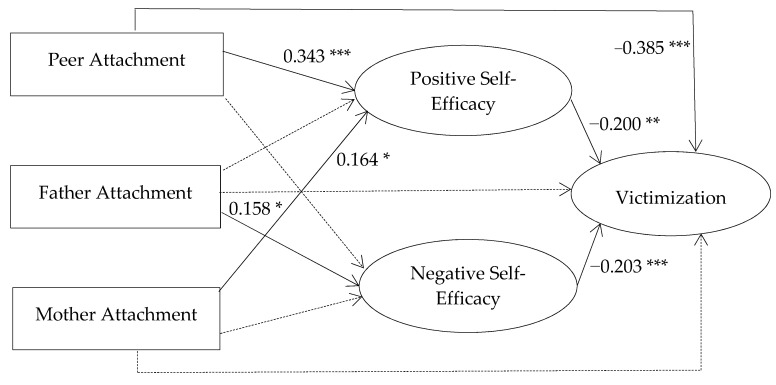
Path model of the relations among peer, father, and mother attachment, Perceived self-efficacy to express positive affect, perceived self-efficacy in regulating negative affect and victimization across youth from Spain. Standardized coefficients are depicted. Self-efficacy positive affect = perceived self-efficacy to express positive affect, self-efficacy negative affect = perceived self-efficacy in regulating negative affect. Significant covariations between peer, father and mother attachment, and between positive and negative self-efficacy are not depicted. * *p* < 0.05; ** *p* < 0.01; *** *p* < 0.001.

The cross-sectional model was analyzed using structural equations modeling. The model captured the relationships between latent and observed variables in the same wave. The latent variables were perceived self-efficacy to express positive affect, perceived self-efficacy in regulating negative affect and victimization. The observed variables were peer attachment, father attachment and mother attachment. The model captured the relationships between peer attachment, father attachment and mother attachment, and victimization through regulatory emotional self-efficacy (perceived self-efficacy to express positive affect, and perceived self-efficacy in regulating negative affect). The direct relationship between attachment and regulatory emotional self-efficacy was also studied, and the relationship of them with victimization. Furthermore, the relationship between regulatory emotional self-efficacy and victimization was also considered. The results indicate a good fit between the model and the empirical data: *χ*^2^ (849) = 1504.027, *p* = 0.000. The following fit indexes were also obtained: CFI = 0.962 and TLI = 0.960. All fit indexes showed a very good fit. Finally, error measurement was calculated: RMSEA = 0.038. Values below 0.10 indicate acceptable error and values around 0.06 indicate a very good fit [46]. Table 4 and Figure 1 show the path values. 

There was a significant direct effect from peer attachment to positive self-efficacy. There was also a significant effect from peer attachment to victimization. Furthermore, there was a significant direct relation from father attachment to negative self-efficacy, and there was also a significant direct relation from mother attachment to positive self-efficacy. Finally, there were both significant direct relations from positive and negative self-efficacy to victimization.

Bias corrected bootstrap confidence intervals suggested that there was a significant indirect effect from peer attachment to victimization via the perception of self-efficacy in expressing a positive affect (*β* =−0.06, *p* = 0.006). There was also a significant effect from father attachment to victimization via perceived self-efficacy in regulating a negative affect (*β* = −0.03, *p* = 0.035).

As we expected, the results showed a positive relationship between peer and parents attachment with emotional self-efficacy which, in turn, was negatively related to victimization.

## 4. Discussion

The main objective of this study was to examine the role of regulatory emotional self-efficacy as a possible mediator in the association between peer and parents attachment and victimization. We hypothesized that peer and parents attachment could influence victimization through regulatory emotional self-efficacy. In our study, peer attachment influenced victimization only through the expression of positive emotions, and father attachment influenced victimization only through the management of negative emotions. However, mother attachment had no influence in victimization through regulatory emotional self-efficacy.

Related to peer attachment, we found that, on the one hand, emotional self-efficacy was a mediator variable between this attachment and victimization, particularly through the expression of positive emotions. In this sense, our study followed the line of other research which demonstrated that positive peer attachment is an important factor in the development of positive emotions, emotional self-regulation and behavior, and it protects from maladaptive behavior like victimization [11,15]. On the other hand, the rejection and expectation of rejection on the part of the peers can place the adolescent in a situation of vulnerability and cloud over positive free-flowing relationships with the environment [47]. At the same time, they can have serious repercussions on health and social adjustment [14,48]. These results follow the line of other recent studies that showed the mediator role of emotional self-efficacy between peers and parents attachment and other internalizing [49] and externalizing variables [37,38]. Also, peer attachment is associated directly and negatively with victimization, which reinforces the importance and the role played by peer attachment in adolescence, as it facilitates experiencing and expressing positive emotions and is associated with lower victimization.

Regarding father attachment, in this study emotional self-efficacy mediates the association between this attachment and victimization. In this sense, previous studies demonstrated that both father attachment and mother attachment were associated with emotional self-efficacy and, in turn the latter mediated in the relation with internalized [37] and externalized symptoms like aggressive behavior and victimization [24,38]. However, in this study, it was the perception that adolescents had about the attachment to their father that influenced victimization through managing negative emotions like anger and distress. The stronger the attachment to the father, the higher the perceived capacity by the adolescent to manage negative affects against threats that arouse anxiety, incite anger, rejection and lack of respect, and against distress and despondency which, in turn, result in a lower victimization.

A possible explanation could be that, traditionally, the relationship style with the father has been more related to the abilities concerning how to manage and handle the affect described, while in the case of the mother, the relationship style with her has been traditionally linked to the expression of kindness and affection to others [50]; to get oneself to express enthusiasm and enjoyment and to feel satisfied with personal achievements.

These findings suggest that regulatory emotional self-efficacy could be one mechanism explaining how peer and parent attachment influences victimization.

Finally, related to mother attachment, we found that mother attachment had no indirect influence in victimization through emotional self-efficacy nor directly. In previous studies, [37] results revealed that, specifically, maternal attachment was associated with internalizing symptoms and this association was fully mediated by regulatory emotional self-efficacy. One possible reason for our results may be that the effect of attachment, affect and communication of the mother on victimization needs the joint action of other emotional variables like empathy, as shown by other studies. These studies confirmed the need to include empathy and emotional regulation among the factors related to psychological and social adaptation and peer attachment. These studies also showed that through empathy, the said effect on victimization was established [33]. These studies showed that the role of parent attachment is important through empathy, which is key in peer attachment and the perceived support of peers. 

In this study, we found that peer and mother attachment was directly and positively associated with the expression of positive emotions and father attachment with the managing of negative emotions. In accordance with this, previous research suggested that individuals with a strong attachment to their father, mother, or peers typically have successful emotional regulation experiences [37]. Moreover, the regulation of positive and negative emotions has a different relationship with social and emotional adjustment. The experiencing and expression of positive emotions is associated with gratifying social relationships, health and career success, while difficulty to regulate negative emotions is associated with problematic interpersonal behavior [51]. 

In conclusion, despite the changes produced during this stage (adolescence) between adolescents and their parents, the role of family is not less important than that of peers [15,33].

The findings provide useful information about the framework of adolescent relationships and the relation of positive emotional variables, such as peer/parent attachment, in order to establish new ways of intervention for successful treatment of maladaptive behaviors (victimization, bullying, aggressiveness) including emotional regulation, negative and positive emotions, and processes to cultivate throughout adolescence. Findings highlight the importance of prevention of victimization to improve the quality of life and health in adolescence and in the complete life cycle. 

In this sense, it is important that intervention programs include strategies that develop or encourage perceived self-efficacy, meaning the beliefs held about one’s own ability to express positive emotions and to regulate negative emotional states when they appear as a response to adverse or frustrating situations.

Research shows that these regulating beliefs of emotional self-efficiency contribute to regulate impulsive tendencies [52,53], as well as low levels of internalization problems [54,55], insofar as they modulate the urgency of emotions and sustain self-regulation mechanisms. 

The results obtained place as a future challenge the analysis of any other variables (emotional, personal, family) that act during adolescence to produce an increase in victimization. Finding no direct or indirect effect of mother attachment on victimization assessed in adolescents, leads us to consider the possibility of analyzing it in interaction with other family variables in prediction of victimization, such as following the line of recent studies on adolescence which, for example, [33] confirm the role of a parenting style defined by affection and communication in relation to victimization. The study could be enriched, too, by using two data sources for all variables: information about adolescents and about parents.

Finally, it is necessary to mention the limitations of our study. First, all variables were obtained using self-reported data. Even though it has been demonstrated that the self-reporting information of adolescents is more reliable, and has higher predictive value than that provided by the families [56], depending less on social desirability problems [57], it is important to take into consideration the contribution of other sources of information for the study.

Second, the data was cross-sectional, so causal relationship could not be established. A longitudinal methodology would contribute greater strength by its rigor in research, especially at ages in during which the subject is developing and important changes are occurring in cognitive, emotional and behavioral processes which influence interaction with other people and psycho-social adjustment.

Third, it is important to analyze the differences of gender in the structure of self-efficacy to control negative emotions and express positive ones [58].

## 5. Conclusions

Results showed direct relations among victimization and the variables peer attachment and emotional self-efficacy in expressing positive emotions, and in managing negative emotions (anger and despondency/distress).

Furthermore, emotional self-efficacy was a mediator variable between attachment and victimization, emotional self-efficacy in expressing positive emotions appeared to mediate the relations between peer attachment and victimization, and emotional self-efficacy in managing negative emotions appeared to mediate the relations between father attachment and victimization. 

When the adolescent perceives strong and safe attachment to peers, the greater the belief in their ability to manage positive emotions which, in turn, produces a decrease in the perception of victimization. In the same way, when father attachment is strong and safe, the greater is the adolescent’s self-perception of being able to manage negative emotions (anger and despondency/distress) which, in turn, has a bearing on lower victimization.

## Figures and Tables

**Table 1:** Characteristics of survey participants.

Variable	Percentage (%)
Gender	
Male	55.5
Female	44.5
Family structure	
Two-parent households	70.8
Divorced parents	23.2
Mother’s educational level	
< secondary school diploma	24
Secondary school diploma	35.4
University education	40.6
Father’s educational level	
< secondary school diploma	30.3
Secondary school diploma	35.2
University education	34.6
Origin	
Spain	89.7
Latin America	8.6
Eastern European countries	1.7

**Table 2:** Items composition and reliability analysis results (Cronbach’s alpha).

Variable	*N* Items	α
1. Victimization	28	0.96
2. Peer attachment	25	0.79
3. Father attachment	25	0.89
4. Mother attachment	25	0.86
5. Self-efficacy positive affect	4	0.74
6. Self-efficacy negative affect	8	0.80

**Table 3:** Descriptives and correlation matrix for victimization, peer attachment, father attachment, mother attachment, self-efficacy positive affect and self-efficacy negative affect.

Variable	1	2	3	4	5	6
1. Victimization	-					
2. Peer attachment	−0.43 **	-				
3. Father attachment	−0.05	0.13 **	-			
4. Mother attachment	−0.02	0.10 *	0.54 **	-		
5. Self-efficacy positive affect	−0.23 **	0.32 **	0.11 **	0.19 **	-	
6. Self-efficacy negative affect	−0.14 **	0.07	0.17 **	0.07	0.17 **	-
Mean	1.29	3.69	3.33	3.45	4.31	3.13
SD	0.51	0.55	0.46	0.37	0.61	0.75

Note: * *p* < 0.05; ** *p* < 0.01; Self-efficacy positive affect = Perceived self-efficacy to express positive affect, Self-efficacy negative affect = Perceived self-efficacy in regulating negative affect

**Table 4:** Path coefficient, *p*-value, and goodness-of-fit of the model.

Path	*β*	*p*-Value
1. Peer attachment → Positive self-efficacy	0.34	0.000
2. Peer attachment → Negative self-efficacy	0.16	0.18
3. Peer attachment → Victimization	−0.38	0.000
4. Father attachment → Positive self-efficacy	−0.04	0.49
5. Father attachment → Negative self-efficacy	0.15	0.02
6. Father attachment → Victimization	0.03	0.62
7. Mother attachment → Positive self-efficacy	0.16	0.04
8. Mother attachment → Negative self-efficacy	−0.04	0.62
9. Mother attachment → Victimization	0.04	0.37
10. Positive Self-efficacy → Victimization	−0.20	0.01
11. Negative Self-efficacy → Victimization	−0.20	0.000
Global goodness of fit indices		
	*χ^2^*	1504.02
	*df*	84
	*p*	0.000
	CFI	0.96
	TLI	0.96
	RMSEA	0.03

## Data Availability

The data presented in this study are available on request from the corresponding author. The data are not publicly available due to are data of a Research Project of Valencian Government
